# Dietary sucrose determines the regulatory activity of lithium on gene expression and lifespan in *Drosophila melanogaster*

**DOI:** 10.18632/aging.205933

**Published:** 2024-06-10

**Authors:** Katharina Jans, Kai Lüersen, Jakob von Frieling, Thomas Roeder, Gerald Rimbach

**Affiliations:** 1 Division of Food Science, Institute of Human Nutrition and Food Science, University of Kiel, Kiel D-24118, Germany; 2 Division of Molecular Physiology, Institute of Zoology, University of Kiel, Kiel D-24118, Germany

**Keywords:** lithium, longevity, glycogen synthase kinase 3, fruit fly, trace element

## Abstract

The amount of dietary sugars and the administration of lithium both impact the lifespan of the fruit fly *Drosophila melanogaster*. It is noteworthy that lithium is attributed with insulin-like activity as it stimulates protein kinase B/Akt and suppresses the activity of glycogen synthase kinase-3 (GSK-3). However, its interaction with dietary sugar has largely remained unexplored. Therefore, we investigated the effects of lithium supplementation on known lithium-sensitive parameters in fruit flies, such as lifespan, body composition, GSK-3 phosphorylation, and the transcriptome, while varying the dietary sugar concentration. For all these parameters, we observed that the efficacy of lithium was significantly influenced by the sucrose content in the diet. Overall, we found that lithium was most effective in enhancing longevity and altering body composition when added to a low-sucrose diet. Whole-body RNA sequencing revealed a remarkably similar transcriptional response when either increasing dietary sucrose from 1% to 10% or adding 1 mM LiCl to a 1% sucrose diet, characterized by a substantial overlap of nearly 500 differentially expressed genes. Hence, dietary sugar supply is suggested as a key factor in understanding lithium bioactivity, which could hold relevance for its therapeutic applications.

## INTRODUCTION

For many, a long and healthy life is a desirable pursuit, so there is an everlasting search for antiaging drugs, also referred to as geroprotectors [1]. Agents such as rapamycin, spermidine, metformin, trametinib and, more recently, lithium have been identified to be effective in animal studies, although their efficacy in humans has not been confirmed [2–6]. However, along with the convenient intake of such substances, these molecules significantly impact lifespan in *in vivo* models [7–9].

The alkali metal lithium is widely used for mood stabilization and it is also known for its neuroprotective, immune-regulatory and survival-promoting properties [4, 6, 10, 11]. Over the past century, various lithium-sensitive enzymes have been identified [12]. These enzymes include glycogen synthase kinase-3 (GSK-3), which was first demonstrated to be inhibited by lithium in embryos of the clawed frog *Xenopus laevis* [13]. Similar to the insulin/PI3K/Akt cascade, lithium decreases the activity of this highly evolutionarily conserved kinase across species [14]. Lithium’s action on GSK-3 suggests a regulatory effect on glucose and energy metabolism, as GSK-3 controls glycogen synthase activity, mitochondrial activity and the respiratory chain [15, 16]. Consistent with this finding, lithium has been suggested to exert insulin-like and antidiabetic bioactivity by multiple research groups [17–20]. Several studies in mammals have shown that lithium promotes glycogen synthesis, glucose tolerance, and insulin sensitivity [18, 21–23]. However, systematic studies on the general influence of this alkali metal on glucose homeostasis and their consequences for organisms are lacking and inconsistent, with the result that this subject is still poorly understood [18].

Li^+^ ions have been suggested to inhibit GSK-3 directly due to the intracellular displacement of Mg^2+^ ions, since both have a similar atomic radius [24]. As a result, the β-arrestin/protein phosphatase 2A/Akt complex is no longer stabilized by GSK-3, facilitating the accumulation of free Akt in the cytosol. The insulin signaling cascade activates non-complexed Akt to phosphorylate and inactivate the two GSK-3 isoforms at either serine 9 (GSK-3β) or serine 308 (GSK-3α), which further promotes the inhibition of GSK-3 kinase activity [19, 24–26].

In general, decreasing the activities of GSK-3 is predicted to benefit various biological processes, such as autophagy, cell survival and cell differentiation [16, 27, 28]. Thereby, it is even believed to counteract the onset of age-related diseases [27]. Thus, via direct and indirect mechanisms, lithium supports the decrease in GSK-3 activity, which has been postulated to benefit health and lifespan [9, 29]. The GSK-3 inhibitor lithium promotes survival in simple model organisms. Lifespan extension due to lithium supplementation was first reported in the nematode *Caenorhabditis elegans* in 2008 and in the fission yeast *Saccharomyces pombe* in 2014 [30, 31]. Only two years later, Castillo-Quan and colleagues discovered that lithium also promotes longevity in the fruit fly *Drosophila melanogaster* when added to a standard 5% sugar/10% yeast/2% agar diet at concentrations of 1 to 25 mM LiCl [29, 32, 33]. This phenomenon was associated with a lithium dose-dependent increase in inhibitory phosphorylation of the GSK-3 homolog Shaggy (Sgg), which led to activation of nuclear factor erythroid 2-related factor (Nrf-2) and subsequent expression of enzymes needed for xenobiotic stress resistance [29]. Yet, whether or not the mechanisms mentioned above account for the increased inhibitor phosphorylation of *Drosophila* Sgg has not been fully established.

Adjustment of the dietary sugar concentration can have a substantial impact on life expectancy in female fruit flies. Compared to low-sugar diets, a moderate isocaloric rise in added sugar of up to ~15 % increasingly promotes the survival of female flies, while extremely high-sugar diets (usually 20-30 % sucrose) drastically shorten the lifespan [7, 34]. Similar findings have also been reported in *ad libitum* fed mice, where median lifespan was the greatest when carbohydrate intake was high and protein intake low [35]. Overall, the geometry of macronutrients appears to be a strong determinant of the lifespan across species. While such findings were assigned to changes in macronutrient supply affecting the nutrient sensing insulin and TOR pathways, the exact mechanism by which dietary sugars influence the lifespan in *Drosophila* has not yet been fully established [7]. It is possible, however, that lithium and dietary sugar both act through the regulation of GSK-3 activities to exert their survival-promoting properties [29, 36]. Based on this proposed overlapping bioactivity of dietary sugar and lithium in the female fruit fly, we decided to investigate the extent of these similarities and whether a joint mechanism lies at their root.

## RESULTS

### Dietary sugars and lithium had a similar impact on lifespan

**Figure 1:**
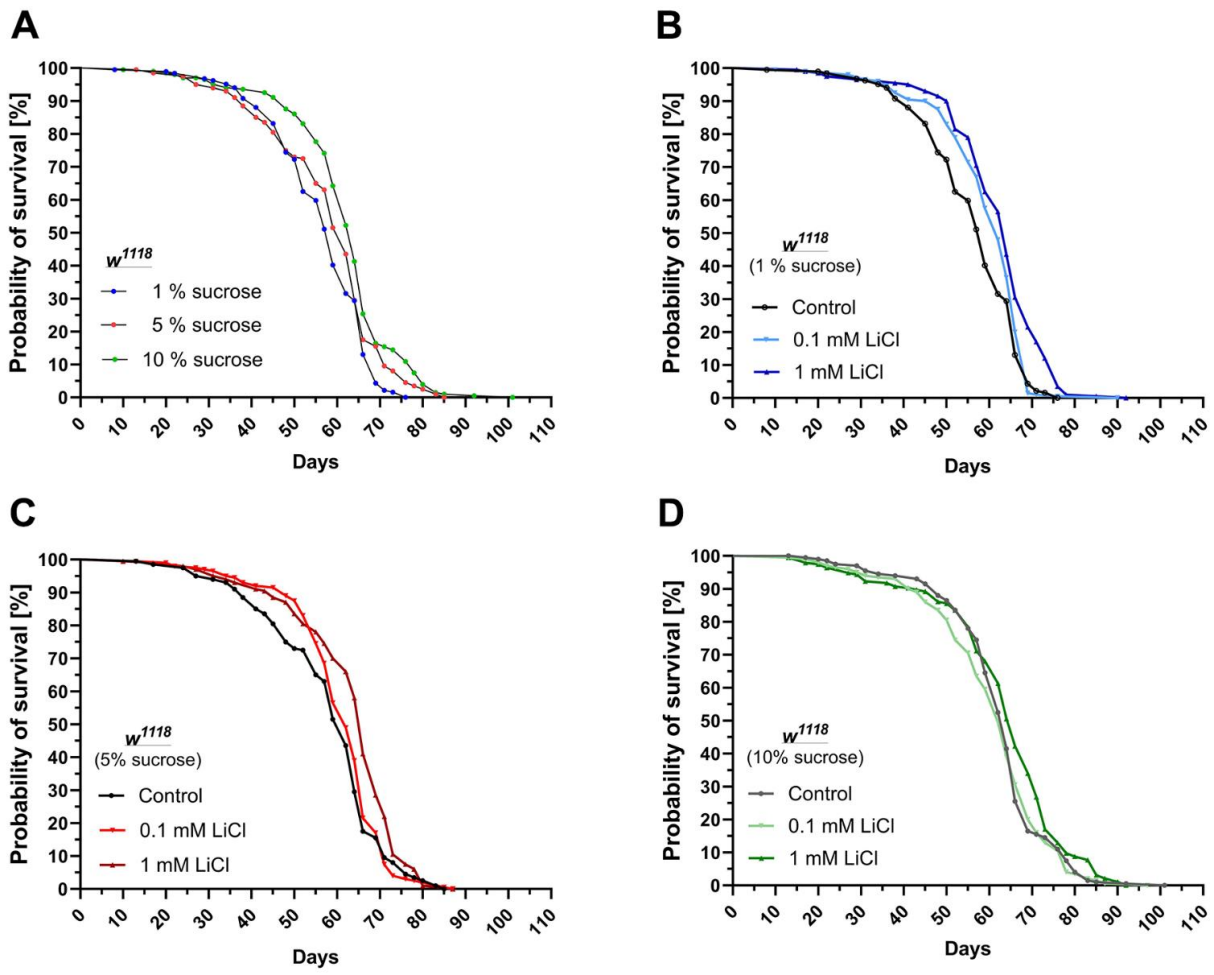
**Lithium and dietary sucrose have a similar impact on the lifespan.** Female *w^1118^* flies were subjected to life-long interventions receiving either the 1%, 5% or 10% sucrose diet supplemented with 0, 0.1 or 1 mM LiCl. (**A**) Increasing the dietary sucrose content to 10% extended the lifespan of the flies. (**B**) Supplementing 0.1 or 1 mM LiCl to the 1% sucrose diet extended the lifespan in a similar manner. (**C**) Supplemented to the 5% sucrose diet, only the dose of 1 mM LiCl significantly improved survival. (**D**) When added to the 10% sucrose diet, the positive impact of lithium on the lifespan further declined. Survival curves were compared using the Log-Rank Mantel-Cox test (p ≤ 0.05).

**Figure 2:**
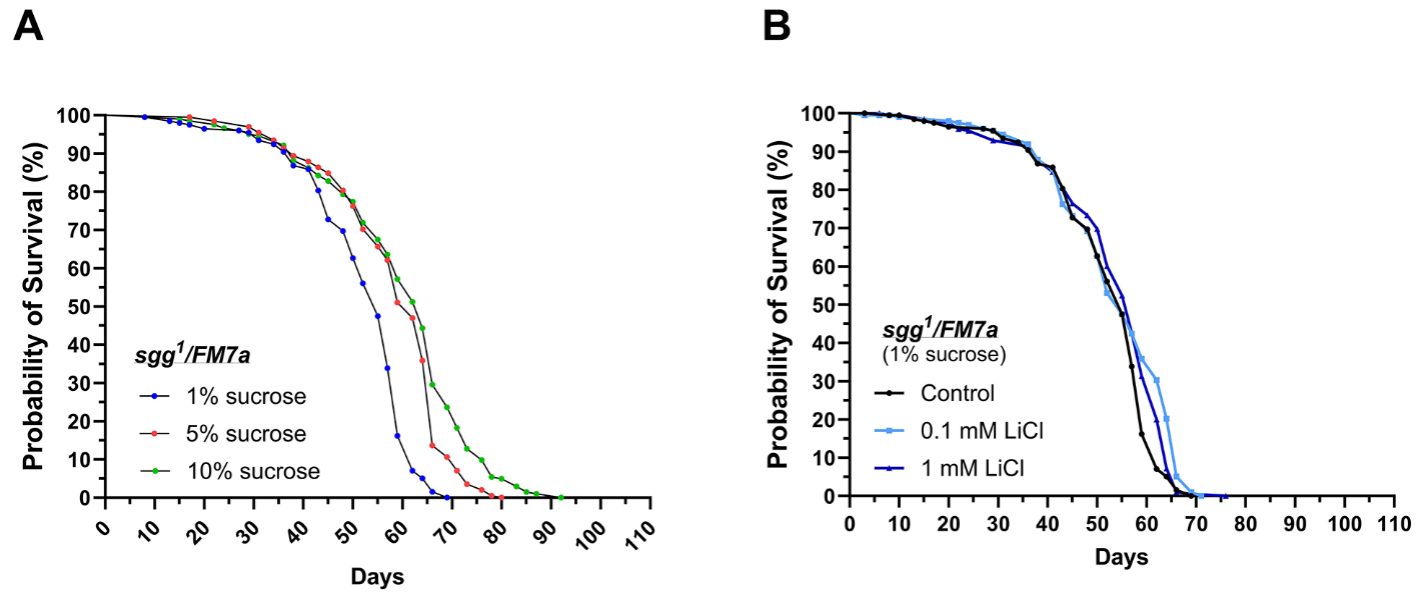
***sgg^1^FM7a* are less responsive to lithium but highly responsive to dietary sucrose in regards of lifespan extension.** Female *sgg^1^FM7a* flies were subjected to life-long interventions receiving either the 1%, 5% or 10% experimental diet supplemented with 0, 0.1 or 1 mM LiCl. (**A**) Dietary sucrose promoted longevity in *sgg^1^FM7a*. (**B**) Supplementing 0.1 or 1 mM LiCl to the 1% sucrose diet mildly promoted survival in *sgg^1^FM7a*. Survival curves were compared using the Log-Rank Mantel-Cox test (p ≤ 0.05).

As shown in Figure 1A and Table 1, elevating the dietary sucrose content from 1% up to 10% improved the median (+5 days) as well as the maximum lifespan (+11 days) in *w^1118^* females. Supplementing 0.1 mM up to 1 mM LiCl to the 1% sucrose diet had a similar impact on the median and maximal lifespan of the flies. The lower dose of 0.1 mM LiCl improved median survival by three days but not the maximal lifespan compared to the non-supplemented low sucrose control diet. The higher dose of 1 mM LiCl improved median survival by five days and prolonged the maximum lifespan by seven days (see Figure 1B and Table 2). When added to the standard 5% sucrose diet, 0.1 mM LiCl had no significant impact on the survival curve, whereas 1 mM LiCl improved median survival by four days and maximal lifespan by only two days (see Figure 1C and Table 1). Lithium added to the 10% sucrose diet only had a small but still significant impact on the survival curve with the higher dose of 1 mM LiCl (see Figure 2D and Table 2).

**Table 1:** Sucrose promotes survival in female *w^1118^*.

**Sucrose [%]**	**LiCl [mM]**	**Median survival [d]**	**Max. lifespan [d]**	**p-value**
1	-	59	71	-
5	-	62	78	**0.01**
10	-	64	82	**≤ 0.0001**

P-values of pairwise log-Rank tests are given (compared to 1% sucrose).

**Table 2:** The impact of lithium on the survival of female *w^1118^* varies depending on the sugar content of the diet.

**Sucrose [%]**	**LiCl [mM]**	**Median survival [d]**	**Max. lifespan [d]**	**p-value**
1	-	59	71	-
1	0.1	62	71	**0.01**
1	1	64	78	**≤ 0.0001**
5	-	62	78	-
5	0.1	62	76	0.20
5	1	66	79	**≤ 0.0001**
10	-	64	82	-
10	0.1	62	81	0.54
10	1	66	86	**0.02**

P-values of pairwise log-Rank tests are given (compared to the non-supplemented control).

It was previously demonstrated that lithium promotes longevity by decreasing GSK-3 activity in *w^1118^* flies. Hence, the experiment was repeated with *sgg^1^/FM7a* females, which carry a strong hypomorphic mutation of the gene *shaggy* (*sgg*) encoding the *Drosophila* homolog of GSK-3 [37]. This approach was performed to determine whether GSK-3/Sgg may be involved in sucrose- and lithium-induced lifespan extension. We found that the median and maximum lifespan was extended in response to elevating the dietary sucrose content from 1% up to the standard level of 5%, similar to what was found in *w^1118^* (see Figure 2A and Table 3). However, lithium only significantly affected the survival curves of *sgg^1^/FM7a* flies when added to the 1% sucrose diet, as shown in Table 4 and Supplementary Figure 1. Adding 0.1 mM LiCl to the 1% sucrose diet extended the maximum lifespan by three days, whereas the higher dose of 1 mM LiCl increased median survival by two days and maximum lifespan by only one day. Accordingly, *sgg^1^/FM7a* flies were non-responsive to lithium when added to diets with higher sucrose content.

**Table 3:** Sucrose promotes survival in female *sgg^1^FM7a*.

**Sucrose [%]**	**LiCl [mM]**	**Median survival [d]**	**Max. lifespan [d]**	**p-value**
**1**	-	55	65	-
**5**	-	59	74	**≤ 0.0001**
**10**	-	64	82	**≤ 0.0001**

P-values of pairwise log-Rank tests are given (compared to 1% sucrose).

**Table 4:** Lithium extends the lifespan in female *sgg^1^FM7a* solely when supplemented to a diet prepared with 1% sucrose.

**Sucrose [%]**	**LiCl [mM]**	**Median survival [d]**	**Max. lifespan [d]**	**p-value**
**1**	-	55	65	-
**1**	0.1	55	68	**0.0004**
**1**	1	57	66	**0.01**
**5**	-	59	74	-
**5**	0.1	59	74	0.66
**5**	1	59	70	0.08
**10**	-	64	82	-
**10**	0.1	64	80	0.39
**10**	1	66	80	0.06

P-values of pairwise log-Rank tests are given (compared to the non-supplemented control).

### Lithium supplementation in a low-sugar diet increased whole-body glucose levels

**Figure 3:**
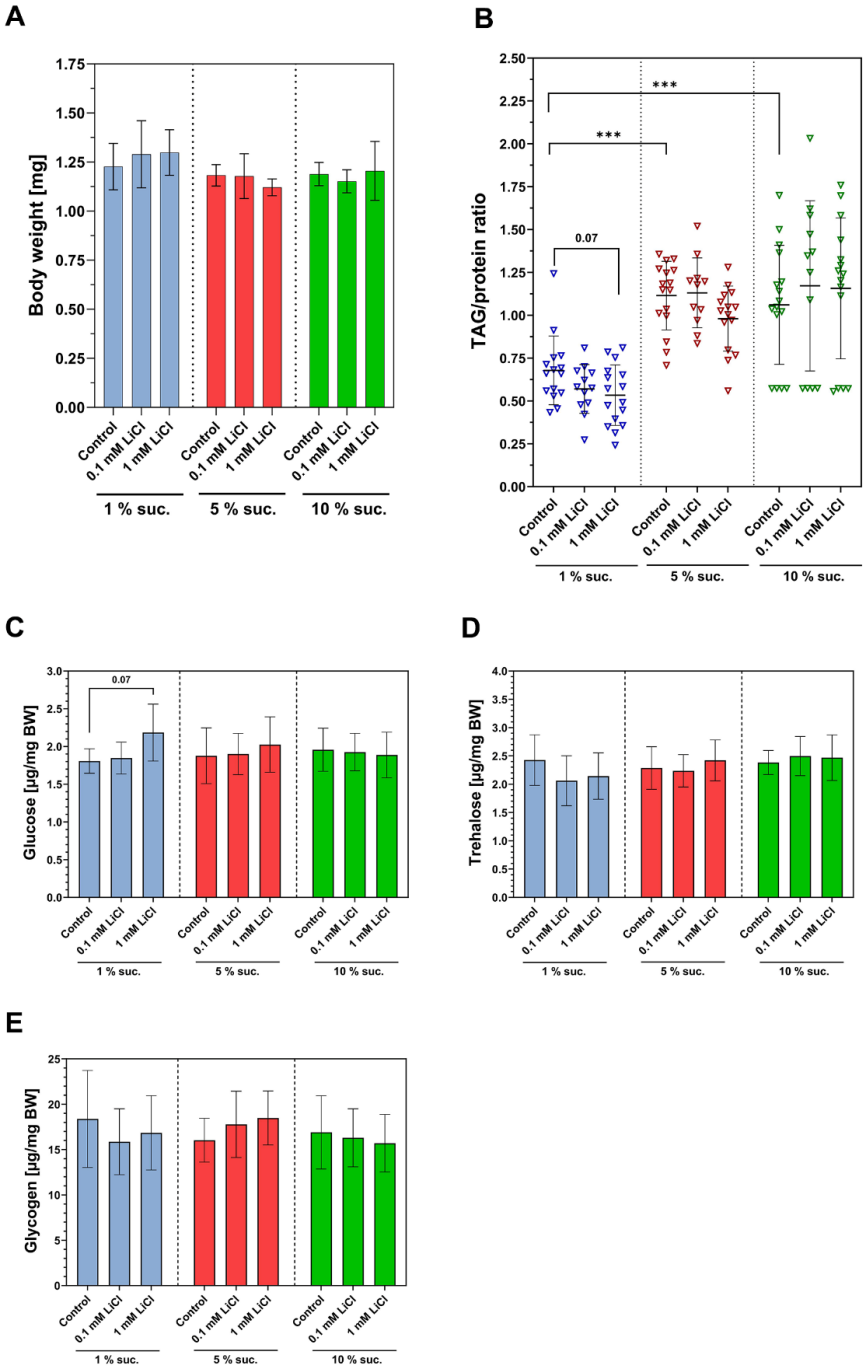
**Lithium and dietary sucrose affect the body composition differently.** Female flies were subjected to a 1%, 5% or 10% sucrose diet supplemented with 0, 0.1 or 1 mM LiCl and the body weight and body composition were examined after seven days of treatment. (**A**) Neither lithium, nor sucrose altered the body composition (one-way ANOVA, p ≤ 0.05). (**B**) Feeding 1% sucrose significantly lowered the TAG/protein ratio compared to a 5% or 10% sucrose diet (Dunnett’s T3 multiple comparisons test, ***p ≤ 0.001). (**C**–**E**) Supplementing 1 mM LiCl to the 1 % sucrose diet slightly increased whole-body glucose level in the fly (one-way ANOVA, p = 0.07). Altering the sucrose content of the diet had no impact on any of the carbohydrate levels in the fly. Bars represent means ± SD. TAG, triacylglycerides; Suc., sucrose; BW, body weight. All data are reported as mean ± SD.

Dietary interventions that promote survival also often affect the body composition of the fruit fly due to adaptations in energy metabolism. Thus, since increasing the dietary lithium supply to 1 mM had a similar impact on the lifespan as elevating the sucrose content from 1% up to 10%, we investigated whether there are also similarities regarding their impact on body composition. Neither lithium nor the sucrose content of the diet had an impact on the body weight of *w^1118^* females after one week of treatment (see Figure 3A). However, as shown in Figure 3B, the TAG/protein ratio was significantly lower in the flies fed the 1% sucrose diet. Moreover, 1 mM LiCl slightly but non-significantly further lowered the TAG/protein ratio when added to the 1% sucrose diet (p = 0.07). Glucose, trehalose and glycogen levels were mostly unaffected by altering the levels of both lithium and sucrose. Only the dose of 1 mM LiCl slightly increased the glucose level of the fly, which was exclusively observed when LiCl was added to a 1% sucrose diet (p = 0.07).

### Dietary sucrose affected the impact of lithium on the GSK-3 phosphorylation rate

**Figure 4:**
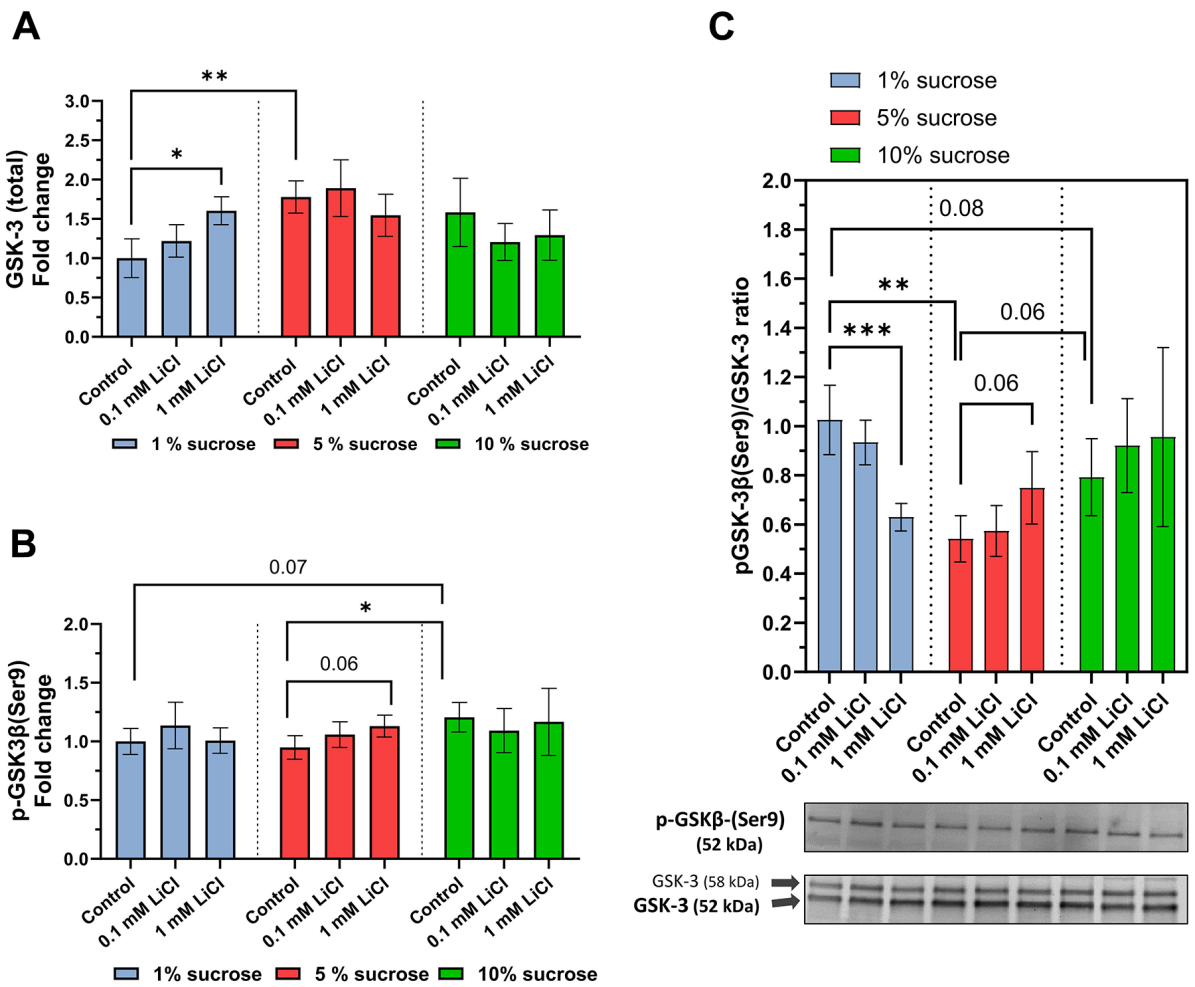
**Dietary sucrose modulates lithium’s influence on the GSK-3 phosphorylation ratio.** Whole-body protein extracts of *w^1118^* females treated with the experimental diet were obtained for Western blot analysis. (**A**) 1 mM LiCl supplemented to the 1% sucrose diet elevated total levels of GSK-3 (Dunn’s multiple comparisons test, *p ≤ 0.05) as did elevating the dietary sucrose content up to 5% (Dunnett’s T3 multiple comparisons test, **p ≤ 0.005). (**B**) Lithium supplemented to the 5% sucrose diet elevated levels of p-GSK-3 similar to the 1% sucrose diet compared to the 5% and 1% sucrose diets (Dunnett’s T3 multiple comparisons test, *p ≤ 0.05). (**C**) Lithium significantly lowers the pGSK-3/GSK-3 ratio when added to the 1% sucrose diet (Dunn’s multiple comparisons test, ***p ≤ 0.001). In contrast, added to the 5% sucrose diet, lithium slightly elevated the pGSK-3/GSK-3 ratio. Co-administered to 10% sucrose, lithium had no significant impact on the pGSK-3/GSK-3 ratio. In general, pGSK-3/GSK-3 ratios of flies treated with the 1% sucrose diet were significantly higher than that of those receiving the 5% and 10% sucrose diets. Two known *Drosophila* GSK-3 isoforms were detected at 52 kDa (G, major isoform) and 58 kDa (SGG39). Overall, feeding the non-supplemented 5% sucrose diet generated the lowest levels of pGSK-3/GSK-3 ratio (Tukey’s multiple comparisons test, **p ≤ 0.005). All data are reported as mean ± SD.

Following addition to a standard diet with medium sugar content, the longevity-promoting effects of lithium have previously been assigned to its potential to promote GSK-3 inhibitory phosphorylation [9, 29]. Therefore, we explored whether changes in dietary sucrose content also affect the GSK-3 phosphorylation rate and, consequently, influence the potential of lithium to inhibit GSK-3. Western blot analysis revealed that the total GSK-3 level of whole-fly extracts was generally the lowest when they were fed the low-sugar control diet (see Figure 4A). However, similar to raising the dietary sucrose content, adding 1 mM LiCl to the 1% sucrose diet significantly increased the levels of total GSK-3 (p = 0.02).

The levels of GSK-3 phosphorylated at the serine 9 residue were slightly increased by adding 1 mM LiCl to the standard 5% sucrose diet compared to the control (p = 0.06) (see Figure 4B). Similarly, elevating the dietary sugar content from 1% or 5% to 10% increased the amount of phosphorylated GSK-3 in the fly. As shown by the pGSK-3/GSK-3 ratio in Figure 4C, however, the influence of lithium on the GSK-3 phosphorylation rate highly depends on the sucrose content of the diet. Surprisingly, the pGSK-3/GSK-3 ratio was highest in the flies fed the 1% sucrose diet and lowest (-50%) in those fed the standard 5% sucrose diet. Added to the 1% sucrose diet, lithium significantly lowered the phosphorylation rate by close to 40% (p = 0.0007). However, when added to the standard 5% sucrose diet, lithium tended to increase the inhibitory phosphorylation of GSK-3 by ~ 20% (p = 0.06), similar to what was previously described by Castillo-Quan and colleagues [29]. However, lithium added to a 10% sucrose diet had no impact on the pGSK-3/GSK-3 ratio.

### Dietary sucrose levels determine the number of genes differentially expressed in response to lithium

**Figure 5:**
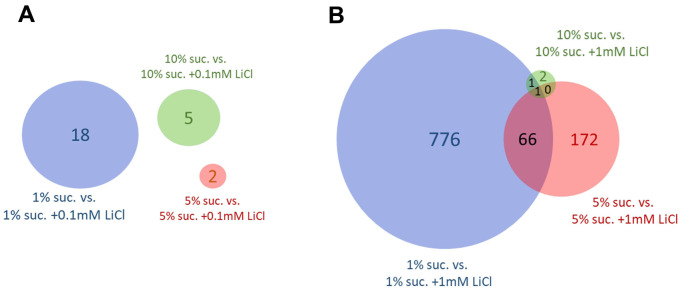
**The dietary sucrose content affects the number of genes differentially expressed by lithium.** Genes differentially expressed (FDR p-value ≤ 0.05) by (**A**) 0.1 mM LiCl or (**B**) 1 mM LiCl supplemented to the experiential diet prepared with either 1%, 5% or 10% sucrose. Overall, the number of genes differentially expressed by lithium in female flies was highest when supplemented to the diet with lowest sucrose content of only 1 %. Suc., sucrose.

Venn diagrams were used to evaluate the impact that changing the dietary sucrose content has on gene expression in response to 0.1 mM or 1 mM LiCl. Figure 5A reveals no overlaps of genes differentially expressed by 0.1 mM LiCl added to either the 1%, 5% or 10% sucrose diet. In general, the impact of this lower lithium dose on the gene expression level was very small. A rather small number of 18 genes responsive to the addition of 0.1 mM LiCl to the 1% sucrose diet was obtained. In Figure 5B, only a single gene, *lithium-inducible factor* (*list)* [38], was found to be significantly regulated by 1 mM LiCl added to all three sugar diets.

### The transcriptional response of the fly to 10% sucrose overlaps with the response to 1 mM LiCl by 50%

**Figure 6:**
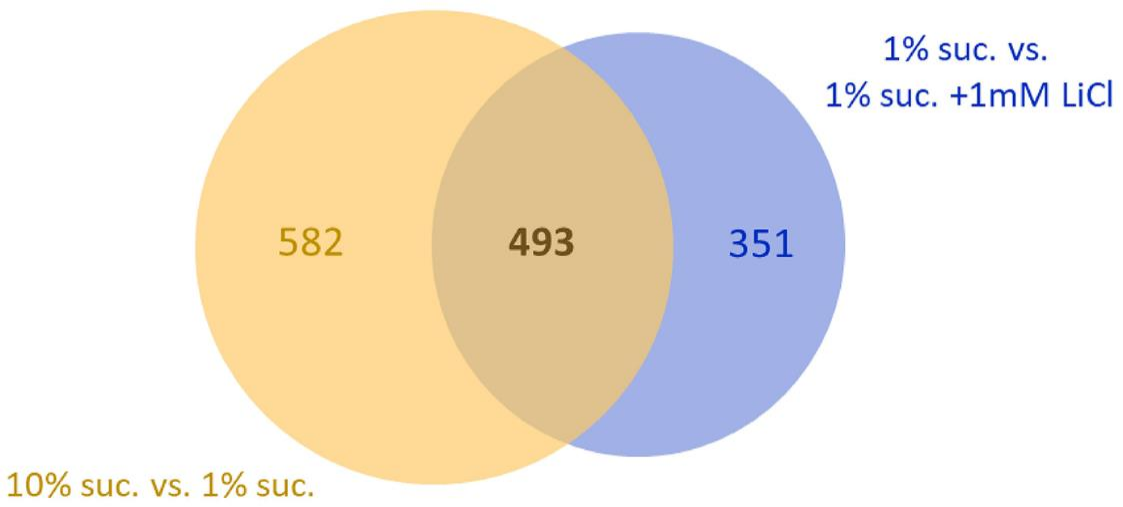
**Supplementing 1 mM LiCl to the 1% sucrose diet mimics half of the transcriptional response to 10% dietary sucrose.** The Venn diagram shows overlaps of two comparisons: 1. the genes whose transcript levels were altered by increasing the sugar concentration from 1% to 10% (yellow circle, total: 1075) and 2. genes whose transcript levels were altered by supplementation of 1 mM LiCl to the 1% sucrose diet (blue circle, total: 844). Both comparisons share an overlap of 493 co-regulated genes differentially expressed in female flies in response to both, sucrose or lithium (FDR p-value ≤ 0.05). Suc., sucrose.

Since we found that lithium and sucrose affect the lifespan of the fruit fly to a similar extent, we chose to investigate whether the two substances also induce similar responses at the transcript level. Again, a Venn diagram (Figure 6) was created showing coregulated genes of two comparisons: (1) the high sugar effect (1% sucrose vs. 10% sucrose) and (2) the lithium effect on the low sugar diet (1% sucrose vs. 1% sucrose + 1 mM LiCl). In total, 493 genes were coregulated by either increasing the dietary sucrose content from 1% to 10% or by adding 1 mM LiCl to the 1% sucrose diet. Accordingly, it was found that lithium copies almost 50% of the fly’s response to high sucrose at the transcript level.

In addition, sucrose- and lithium-responsive coregulated genes were screened for overrepresented transcription factor-binding sites (see Supplementary Table 2.). The analysis revealed that among these 493 genes, binding sites of the transcription factor mothers against decapentaplegic (Mad) were detected with the highest statistical significance (p = 3.54 * 10^-16^).

The DAVID (Database for Annotation, Visualization and Integrated Discovery) tool ranks functional categories based on co-occurrence in a list of genes to disclose biological processes with which they are associated. The results of this analysis are found in Supplementary Table 3. Functional annotation of our coregulated genes revealed significant matches with a variety of different terms, including “chorion” (61.7% match), “immunoglobulin-like fold” (11.2% match), “vision” (11.2% match), “epidermal growth factor (EGF)-like domain” (7.4% match) and “lysosome” (1.5% match).

### Modulation of dietary lithium and sucrose levels affected feeding rate and lithium status

Feed intake is a challenging and fundamental issue in the work with model organisms especially in the context of longevity studies [39–41]. In response to modifying the geometry of the macronutrients, the feed intake of the fly can vary substantially [42]. Therefore, we recorded *w^1118^* feed intake exposed to the different experimental diets in order to detect possible differences in nutrient intake that may have affected the outcome of experiments presented in this work. As shown in Supplementary Figure 3A, with 621 – 763 nL/d, the feed intake of *w^1118^* females was generally the highest when feeding 1% sucrose-containing diets. Among the experimental groups treated with 10% sucrose diets, the feed intake was only 356.7 – 410.6 nL/d. Accordingly, elevating the sucrose content of the diet lowered the total daily feed intake and thereby also the calculated lithium intake of the fly (Supplementary Figure 3B), respectively. This finding raised the question of whether lithium is generally most effective in combination with low sugar because food and thus lithium intakes are higher in these groups. Therefore, the fly’s lithium levels were determined via ICP-MS (see Supplementary Figure 3C). Here, however, a contrasting picture can be observed: the lithium status appears to be almost independent of the differences in feed intake. For the most part, a higher lithium status was observed in animals fed 10% sucrose. Thus, differences in lithium intake do not seem to directly influence the fly’s lithium status and do not explain the increased lithium responsiveness of the fly in terms of longevity or at the transcript level under low sucrose conditions. Instead, the bioavailability and elimination of lithium may be related to the sugar content of the diet. For instance, Li^+^ transport across cell membranes is linked to mechanisms of sodium homeostasis [43, 44]. Therefore, changing the dietary sugar content may as well have an impact on lithium accumulation rates due to a potential dependence on, for example, sodium-dependent glucose cotransporters [45]. In addition, sugar intake was calculated from the food intake data (see Supplementary Figure 3B). Despite the higher food intake of flies fed 1% sucrose, the amount of sucrose consumed was still significantly higher in flies fed with diets containing 10% sucrose. Notably, however, supplementing the 1% sucrose diet with 1 mM LiCl resulted in higher feed intakes of +141.8 nL/d than in the non-supplemented 1% sucrose control. This result was not observed with lithium supplementation of the 10% sucrose diet.

## DISCUSSION

In the present study, we found that in female *D. melanogaster,* the life-prolonging effect of dietary lithium is dependent on the actual sucrose content of the medium. Consistent with previous reports [41, 42], increasing the sucrose content of the *Drosophila* diet from a low level of 1% to a moderate value of 10% prolonged the lifespan of the female fruit fly. Moreover, supplementation of relatively low lithium doses (0.1–1 mM LiCl) proved to be most effective regarding lifespan extension when lithium was added to the low-sucrose diet, thus phenocopying to a large extent the effect of a moderate increase in dietary sucrose. However, when added to a diet containing 10% sucrose, the additional positive impact of 1 mM LiCl on the already improved median and maximum lifespan was considerably smaller. In *Drosophila*, geroprotective drugs, which act via distinct mechanisms, have the potential to exert synergistic effects in terms of longevity. For instance, a triple drug combination of the GSK-3 inhibitor lithium, TOR-inhibitor rapamycin and mitogen-activated protein kinase kinase-inhibitor trametinib produces the greatest lifespan extension compared to single or double administration [9]. However, lithium and dietary sucrose were found to have little to no additive effect on survival when coadministered, indicating that they are more likely to act via the same mechanism. Thus, we determined that sucrose and lithium may promote longevity through a mutual mechanism, which was likely to be GSK-3 inhibition. However, Western blot analysis yielded an unexpected result. Notably, feeding only 1% sucrose resulted in the highest GSK-3 phosphorylation rate and adding lithium increased total levels of GSK-3, resulting in substantially higher rates of non-phosphorylated, presumably active GSK-3. In other words, longevity induced by lithium added to a low sucrose diet was accompanied by an increase in non-phosphorylated GSK-3 levels, which is the exact opposite of what was initially expected. Nevertheless, it must be considered that inhibitory phosphorylation of GSK-3 solely mirrors the secondary indirect mechanism by which the alkali metal is known to downregulate GSK-3 activity [46]. Here, we do not cover lithium’s potential of Mg^2+^ displacement as the direct mechanism of GSK-3 inhibition. Therefore, calculating p-GSK-3/GSK-3 ratios does not give exact information on the activity status of the kinase. Instead, determining the activities and phosphorylation state of GSK-3 substrates would be helpful to gain more information on the full potential of lithium as a GSK-3 inhibitor during low or high sucrose conditions. Taken together, these findings indicate that the survival-promoting properties of lithium during low sugar availability are not achieved through inhibitory GSK-3 phosphorylation.

Various studies support the survival-promoting effect of lithium to be dependent upon sugar availability. For instance, in budding yeast, it was recently found that a comparatively high dose of 200 mM LiCl promotes long-term survival solely upon glucose deprivation (0.5% glucose). However, when the glucose supply was elevated fourfold, lithium induced dose-dependent growth inhibition and decreased long-term viability [47]. Likewise, a year before the work of Castillo-Quan et al. [29], another study was published investigating the effect of lithium on the lifespan of *w^1118^* flies. Here, supplementation with 1, 10 or 20 mM LiCl did not improve the lifespan of male or female flies. However, one notable result is the high median survival among the control groups (84 days in females and 67 days in males). Furthermore, the experimental diet used in this study provided high proportions of different carbohydrate sources (5% corn meal, 3% brown sugar, 7.25% white sugar), yet comparatively low amounts of yeast (2.5%) as a major protein source [48]. Accordingly, apart from the other carbohydrates, the total proportion of added sucrose from white and brown sugar was almost double the 5% sucrose that we consider standard, or which was also used by Castillo-Quan and colleagues [29]. It is therefore possible that the authors did not record the lifespan-prolonging effect of lithium in *w^1118^* flies as their experimental diet did not provide the needed nutritional geometry for the effect to show. In contrast, lithium even reduced the already largely maximized lifespan but only in females, diminishing the female advantage in lifespan [48]. The origin of this observation was not elucidated by the authors but might be relevant to understanding the reliance of lithium’s bioactivity on the ratio of macronutrients in the fly diet. Our Western blot analysis showed that lithium had no effect on the already elevated GSK-3 phosphorylation ratio when added to the 10% sucrose diet. Accordingly, provided that lithium’s indirect mechanism of GSK-3 phosphorylation can be directly associated with its survival-promoting properties, the lifespan should remain unaffected by lithium added to high sucrose diets, as was also confirmed by our own data. Hence, all of these findings support our hypothesis that the effect of lithium on lifespan is dependent on the dietary proportions of sugars, which underlines the relevance of standardization and targeted choice of experimental diets in model organisms.

As mentioned earlier, a decrease in GSK-3 activity has been considered a potential therapeutic treatment for many age-related and metabolic diseases, including cancer, cardiovascular disease, neurodegeneration and even diabetes [16, 49]. Moreover, it has previously been found that decreased GSK-3 activity can improve glucose or lipid metabolic disorders in zebrafish liver cells as well as in an *in vivo* model [50]. In this study, we observed that the body composition of the fly was affected differently by lithium and sucrose. Elevating the dietary sucrose administration shifted the TAG/protein ratio upwards, indicative of increased accumulation of body fat. This finding is in accordance with the literature, as an excess of dietary sugar is used to build up lipid stores [34, 51]. In contrast, lithium lowered the TAG/protein ratio and instead elevated glucose levels of the fly but, yet again, only when added to the 1% sucrose diet. Lithium has previously been shown to dose-dependently lower TAG storage in female flies [29]. Considering these two findings, the lowered TAG/protein ratio and the slightly increased glucose levels, it is likely that lithium mobilizes lipid storage to drive gluconeogenesis but only if the dietary sucrose supply is genuinely low. In the fruit fly, there is negative correlation between storage lipids and lifespan [52], meaning that the influence of lithium on survival is likely related to its regulatory impact on energy metabolism and body composition. However, it is evident that sucrose and lithium induce different shifts in terms of body composition and, therefore, most likely also in regards to macronutrient and energy metabolism. In fact, it was previously shown that 1 mM LiCl sufficiently blocks a high-sugar diet (20% sucrose)-induced *Drosophila* model of hypertriglyceridemia, which further underlines the opposite actions of the two substances in this context [29]. Although inhibition of GSK-3 is predicted to stimulate glycogen synthesis, no changes in glycogen levels were observed in response to either lithium or sucrose in this work. Again, our findings support the data published in 2016 by Castillo-Quan and colleagues, who observed a trend but no significant increase in whole-body glycogen levels even when adding much higher lithium doses of up to 75 mM LiCl to a 5% sucrose diet [29]. The fact that glucose, trehalose, and glycogen levels were largely unaffected by a change in dietary sucrose of 1% to 10% in our study indicates that glucose homeostasis in the fly copes perfectly with fluctuations of this range. Thus, a moderate increase in dietary sucrose of up to 10% promotes survival and does not induce dysregulation of carbohydrate metabolism, as previously demonstrated in a 30% high-sucrose-induced fly model of obesity [34]. We acknowledge that the feed and sucrose intake of some experimental groups varied significantly. For example, a higher feeding rate of the flies receiving the 1% sucrose diet will have increased factors such as the protein intake of the animals, which may also have impacted the parameters considered here, such as survival and body composition. Consequently, it cannot be fully ruled out that some of our observations are partly related to differences in feed intake.

Notably, studies in rodents and humans have previously sparked discussion on whether lithium may play a regulatory role in the maintenance of glucose homeostasis. In patients suffering from bipolar disorder, lithium administration was linked with improved glucose tolerance, whereas discontinuation of the treatment entails the loss of this benefit [53]. Furthermore, lithium has been reported to have neuroprotective properties in Alzheimer’s, Huntington’s and Parkinson’s disease [11, 54]. Disruptions of neuronal glucose metabolism as well as impaired glucose supply of brain cells are discussed to play a major role in neurodegenerative diseases. Thus, considering the impact of lithium on glucose metabolism as a potential driver for these findings may be useful [55]. In Chinese hamsters, lower levels of lithium in the liver, kidney, and muscle were associated with insulin resistance in these tissues, suggesting a possible biological function of lithium in glucose metabolism in rodents [23]. Nevertheless, there are also case reports linking lithium treatment with hyperglycemia, which demonstrates that evidence on this topic is rather inconclusive [18]. Regardless, changes in whole body-glucose levels and GSK-3 phosphorylation dependent on dietary sugar administration are likewise indications for lithium to interfere with carbohydrate metabolism in the fly.

In line with our previous findings, the number of genes differentially expressed in response to lithium was substantially higher when lithium was added to the 1% sucrose diet. Hence, in the fruit fly, lithium exerts the strongest transcriptional response when coadministered with low-dietary sucrose, as was the case for lifespan, GSK-3 phosphorylation rate and body composition. Remarkably, nearly 50% of lithium-responsive genes were also coregulated in response to elevating the dietary sucrose content to 10%. This finding further supports the idea that lithium and sucrose act, at least to some extent, through a joint mechanism that benefits survival in *Drosophila*. Functional annotation revealed significant clustering among these mutually regulated genes assigned to the term “chorion”, which refers to the outer shell of an insect’s egg. Synthesis of the chorion is an important step in *Drosophila* egg production starting as early as mid-oogenesis [56]. This finding is consistent with our previously published data, which are related to the impact of lithium on oogenesis in *Drosophila*. In this study, feeding 1 mM LiCl also affected the transcript levels of eggshell-associated genes in *w^1118^* ovaries [57]. However, the regulation of genes involved in EGF signaling is more likely to be linked to lithium-induced longevity. Often associated with tumor development in mammals*,* the EGF pathway interacts with cell differentiation and cell fate-determining signaling pathways, such as PI3K/Akt, mitogen-activated protein kinase and Jak/Stat [58]. In *Drosophila*, EGF regulates health- and longevity-related processes such as cell differentiation, cell-cell adhesion and autophagy-related processes [59–61]. In total, 24 genes encoding proteins with immune globulin-like domains were found to be upregulated in flies treated with either 1 mM LiCl or 10% sucrose. Some of these genes assigned to EGF and immunoglobulin-like fold are also functionally associated with cell adhesion (alterations in mRNA level of the respective genes associated with all three terms can be found in Supplementary Figure 2). Adhesion proteins such as cadherin are crucial for stem cell maintenance as well as for cell-cell communication and for the binding of pathogens [58, 62]. In cancer research, higher levels of glucose have been shown to increase EGFR/Stat signaling, thereby upregulating the expression of genes involved in pro-mitogenic activities [58]. Thus, it is possible that our high-sucrose diet and low-dose lithium supplementation of 1 mM LiCl have a similar impact on EGF cell signaling, which could have affected the lifespan.

Beyond that, analysis of transcription factor-binding site motifs revealed Mad to be a likely candidate that could be responsible for a substantial proportion of the coregulated genes from the overlap. In fact, Mad, or Smad proteins as mammalian homologs of the *Drosophila* Mad protein, was found, ranking among the list of identified GSK-3 substrates [63]. In *D. melanogaster*, it was found that GSK-3 phosphorylates Mad, initiating its degradation, a mechanism that controls self-renewal and asymmetric division of stem cells [64]. Thus, GSK-3 is a negative regulator or Dpp/Mad signaling in the fly, which makes Mad a valid candidate to be indirectly targeted by lithium, similar to what has been proposed for Nrf-2 and β-catenin [14, 26, 29]. Likewise, GSK-3 inhibits BMP/Smad1 signaling in mammals, which is thought to play a crucial role in, for instance, embryonic pattern formation [58]. Moreover, in *Drosophila* embryos, Dpp/BMP signaling was found to be sensitive to maternal glucose availability. It was demonstrated that embryos of females fed a no-sugar diet had significantly higher counts of pMad-positive nuclei, indicating higher Dpp/Mad activities in response to maternal sugar deprivation [65]. In addition, some mutations in the *Drosophila mad* gene were found to induce female sterility, sensitivity to heat stress, abnormal immune response, and decreased body size and cell count [66–70]. *Mad* RNAi flies are short lived and display alterations in lysosomal maturation and immunity, granting Mad a putative role in the lifespan extension observed in the present study [68, 71, 72]. There is also evidence on crosstalk of BMP/Dpp and EGF signaling in *Drosophila* as well as in mammals, which is why the outcomes obtained here from functional annotation and Pscan analysis could be related in some way [73].

Moreover, it is important to recognize that all observation made in the present study when feeding lithium and sucrose both selectively and in combination are demonstrated only in female flies. Therefore, we acknowledge that the data may have yielded different outcome had males been used. For instance, potential sex-specific effects have been reported it terms of neurotoxicity in patients receiving lithium therapy and lithium was also found to alter estrogen receptor expression [74–76]. At this point, we would like to declare to have carried out the experiments with females as lifespan-extending effects of lithium have previously been recorded only in female flies [29]. It would therefore be interesting to investigate whether or not our findings are also true in males and whether the same dose-response is given, since male flies exhibit a significantly lower feed intake compared to mated females [77].

In overall terms, we found that the bioactivity of lithium in regard to survival, body composition, inhibitory phosphorylation of GSK-3 and the transcriptional response was highly reliant upon the sucrose concentrations of the experimental diet. This observation should be considered in future studies investigating the bioactivity of lithium as a trace element and its pharmacodynamics in *D. melanogaster*. In line with the results of previous studies, we propose that further research is needed to gain a better understanding of how macronutrients influence the bioactivity of lithium and *vice versa*, not only regarding its impact on lifespan but also in terms of its other verified bioactive traits, including mood stabilization, neuroprotection and determination of cell fate [46, 78, 79]. It is also of interest to investigate whether the observed lithium-sugar interaction applies to humans which in turn could be of clinical relevance.

## MATERIALS AND METHODS

### Fly lines and cultivation

The following fly strains (purchased from Bloomington Drosophila Stock Center, Indiana, USA) were used in this study: *w^1118^* (#5905) and *sgg^1^/FM7a/Dp(1;2;Y)w^+^*(#4095). All fly strains were cultured as previously described [57]. Details on the strains, chemicals and other products used in this study can be found in Supplementary Table 1.

### Preparation of the experimental diets

The experimental fly diet was prepared with 10% inactive dry yeast (Genesee, USA), 2% *Drosophila* agar type 2 (Genesee, USA) and either 1%, 5% or 10% sucrose (Carl Roth, Germany) to obtain low, standard and high sugar diet. Diets were also prepared with 0.3% propionic acid (≥ 99.5%, Carl Roth, Germany) and 15 ml of a 20% nipagin (Genesee, USA) stock solution (prepared with ≥ 99.8% EtOH absolute, VWR, USA [w/v]) as preservatives. From each of these three diets, a non-supplemented control diet, a diet supplemented with 0.1 mM LiCl and a diet supplemented with 1 mM LiCl were prepared. For this purpose, 5 M LiCl (Merck, Germany) was diluted in ddH_2_O, and the appropriate volumes were added to the diets. The higher dose of 1 mM LiCl (equal to 6.9 μg Li/L) was chosen based on results previously published by Castillo-Quan et al. [29]. Apparently, this concentration represented the lowest dose tested, which significantly promoted longevity when added to a standard 5% sugar diet. The lower dose of 0.1 mM LiCl (equal to 690 μg Li/L) was chosen based on the average concentrations naturally found in most foods and beverages, which is expected to be between 0.2 μg/kg and 3.4 mg/kg (equal to 1.4 μM - 0.7 mM Li) [80, 81].

### Lifespan experiments

Three-day-old mated female flies of the *w^1118^* or *sgg^1^/FM7a* fly lines were subjected to lifelong treatments with either the low-, medium- or high-sugar diet supplemented with or without 0.1 or 1 mM LiCl. Flies were transferred to fresh medium every other day. Dead flies were counted to calculate the median and maximum lifespan (mean age of the last 10% survivors) and to compare survival curves by means of the log-rank (Mantel-Cox) test.

### Protein extraction and Western blotting

Female *w^1118^* flies were administered the appropriate diets for one week until whole-body protein samples were generated. For this purpose, ten flies per sample were homogenized in 200 μL of RIPA lysis buffer (prepared as previously specified [57]) at 25 Hz for 7 min (TissueLyser, Qiagen, Hilden, Germany). Samples were incubated on ice for 30 min and thoroughly vortexed every 5 min. Next, the samples were centrifuged at 16,000 × g and 4° C for 20 min, and the protein contents of the supernatant were determined using the Pierce BCA Protein Assay Kit (Thermo Fisher Scientific, Germany). The volumes needed for loading 40 μg/well were calculated and added to 5 μL of reducing ROTI load loading dye (Carl Roth, Germany). The samples were heated at 95° C for 5 min and transferred to 10-well 4-15% polyacrylamide precast gels (Bio-Rad Laboratories, USA). Electrophoresis was performed at 80 V within the first 10 min to allow even migration of proteins into the gel and continued at 120 V for an additional 60 min. Next, the gels were exposed to UV light for 7 min. The trans-blot turbo transfer system (Bio-Rad Laboratories, USA) was used to transfer proteins from the activated gel onto a methanol-treated blotting membrane. The UV protein load was quantified, and membranes were blocked using 3% BSA (Carl Roth, Germany) in PBS/0.02% Tween 20 (Sigma-Aldrich, Germany) for 2 h before the primary antibody was added overnight incubation at 4° C. The membranes were washed three times for 5 min with 0.02% Tween 20 in PBS and incubated with the secondary antibody diluted in 3% BSA in PBS/0.02% Tween 20 for one hour at room temperature. The membranes were washed again three times for 10 min and developed using SuperSignal Western Blot Substrate (Thermo Fisher Scientific, USA). For each approach, phosphorylated protein levels were first determined, followed by the determination of total protein. With Image Lab 5.0 (Bio-Rad Laboratories, USA), the signal intensities of the bands were normalized to the UV protein load, and the ratios of phosphorylated/total protein were calculated.

The following primary antibodies were used in this study: phospho-GSK-3β (Ser9) (D85E12) XP (1:500, Cell Signaling Technology, USA, #5558) and anti-GSK-3 clone 4G-1E (1:500, Merck, Germany, #05-412). The following secondary antibodies were used: Immun-Star goat anti-mouse (GAM)-HRP conjugate (1:4000, Bio-Rad Laboratories, USA, #1705047) and Immun-Star goat anti-rabbit (GAR)-HRP conjugate (1:4000, Bio-Rad Laboratories, USA, #1705046).

### Colorimetric analysis of body composition and detection of the lithium status

Female *w^1118^* flies were given the different experimental diets for a week before being starved on 2% agar for 1.5 h to ensure intestinal cleansing. The body weight was determined using a precision scale, with calculation of the average weight of ten flies weighed at once. Then, 500 μL of PBS/0.05% Triton X-100 (Merck, Germany) was added, and the samples were homogenized at 25 Hz for 6 min. The homogenates were centrifuged for 5 min at 14,000 ×g and 4° C. For protein quantification, the untreated supernatant was used. Protein concentrations were calculated again using the Pierce BCA Protein Assay Kit according to the manufacturer’s protocol. Prior to quantification of the triacylglyceride (TAG), glucose, trehalose and glycogen contents, 200 μL of each sample was heat-inactivated for 10 min at 75° C. TAGs were determined via a coupled colorimetric assay according to Hildebrandt et al. [82] using the Infinity triglycerides liquid stable reagent (Thermo Fisher Scientific, Germany). The glucose oxidase/peroxidase-linked (GOD-PAP, Dialab, Austria) kit was used for quantification of all three carbohydrates. For quantification of glucose, 20 μL of the heat-inactivated sample was added to 200 μL of reagent solution. To determine the trehalose content, initially, we added prediluted trehalase (Megazyme, Ireland) in PBS (1:20) to the heat-inactivated samples at a 1:1 ratio and incubated the samples overnight at 37° C. For glycogen quantification, 20 μL of the samples were briefly mixed in a 1:1 ratio with 0.016 U/mL amyloglucosidase (Megazyme, Ireland) prediluted in PBS was also left overnight incubation at 37° C. For a standard curve, glucose (5–80 mg/ml, Dialab, Austria), trehalose dihydrate (10–160 mg/ml, Carl Roth, Germany) and glycogen (10–160 mg/ml, Sigma-Aldrich, Germany) were used. The latter two standards also underwent the appropriate enzyme treatment. For calculation of trehalose and glycogen concentrations in each sample, the amount of glucose measured in undigested samples was subtracted. All samples were incubated for 10 min at 37° C, and absorbance was measured at 450 nm. In order to capture potential differences regarding the lithium status among treatment groups, flies were treated, starved and weighed as described above. The lithium content of each sample (n=50 flies pooled) was determined via inductively coupled plasma mass spectrometry ICP-MS at SYNLAB (Jena, Germany) as previously described [57, 83].

### RNA isolation

Three-day-old synchronized female flies of the *w^1118^* strain were treated with the appropriate experimental diets for one week until RNA was extracted from whole-body lysates. For this approach, ten flies per sample were added to RNase-free cups containing 500 μL of peqGOLD TriFast (VWR, Germany) and homogenized for 4.5 min at 25 Hz. Thereafter, RNA was extracted according to the peqGOLD TriFast manufacturer’s protocol, and RNA concentrations were adjusted to 120 ng/μL using DEPC-treated (Merck, Germany) water. Prior to sequencing at the Institute of Clinical Molecular Biology (IKMB, Kiel, Germany), samples were subjected to DNase treatment using the DNA-free DNA Removal Kit (Thermo Fisher Scientific, Germany) according to the manufacturer’s protocol, and samples were readjusted to 100 ng/μL.

### RNA sequencing and data analysis

The NovaSeq 6000 Sequencing System (Illumina, USA) was used to perform cluster generation and sequencing of mRNA samples, generating read lengths of 2 x 50 bp. For library preparation, the TruSeq Stranded mRNA Prep kit (Illumina, USA) was used. Next, paired-end reads were imported to the CLC Genomics Workbench version 9.5.2 (Qiagen, Germany) and aligned to the *D. melanogaster* BDGP6 (Berkeley *Drosophila* Genome Project release 6) reference database, and failed reads were removed. Differential gene expression analysis was performed, and Venn diagrams were created to catch overlaps of different comparisons. The online bioinformatic Database for Annotation, Visualization and Integrated Discovery (DAVID) tool was applied to generate functional annotation charts. Heat-maps were generated using GraphPad Prism 10.0.2. For identification of potential transcription factors with overrepresented binding site motifs in sequences of coregulated genes obtained from differential gene expression analysis, the online tool Pscan Web 1.6 [84] was employed (database: JASPAR 2020_NR [85]; promotor region specified at transcription start site -450 to + 50). Matrix IDs with p-values < 0.05 were considered overrepresented.

### Statistical analysis

Unless otherwise stated above, all statistical tests were performed using Graph Pad Prism 10.0.2. If not stated otherwise in the caption, data are presented as the mean ± standard deviation (SD). Multiple comparisons were performed to analyze statistical significance regarding lithium- or sucrose-associated effects and p < 0.05 was set as threshold for statistical significance. For this purpose, the data were analyzed for normal distribution (Shapiro-Wilk test) and homogeneity of variance (Kruskal-Wallis test for non-parametric data or Brown-Forsythe test for parametric data), and multiple comparisons of experimental groups were performed based on the outcomes.

## Supplementary Material

Supplementary Figures

Supplementary Tables
