# Supplementary material for: Dietary sucrose determines the regulatory activity of lithium on gene expression and lifespan in Drosophila melanogaster
Source: Aging (Albany NY). 2024 Jun 10;16(11):9309–33. doi: 10.18632/aging.205933 (PMC11210232; doi:10.18632/aging.205933)
Supplement: Supplementary Figures [file aging-16-205933-s001.pdf]

SUPPLEMENTARY FIGURES

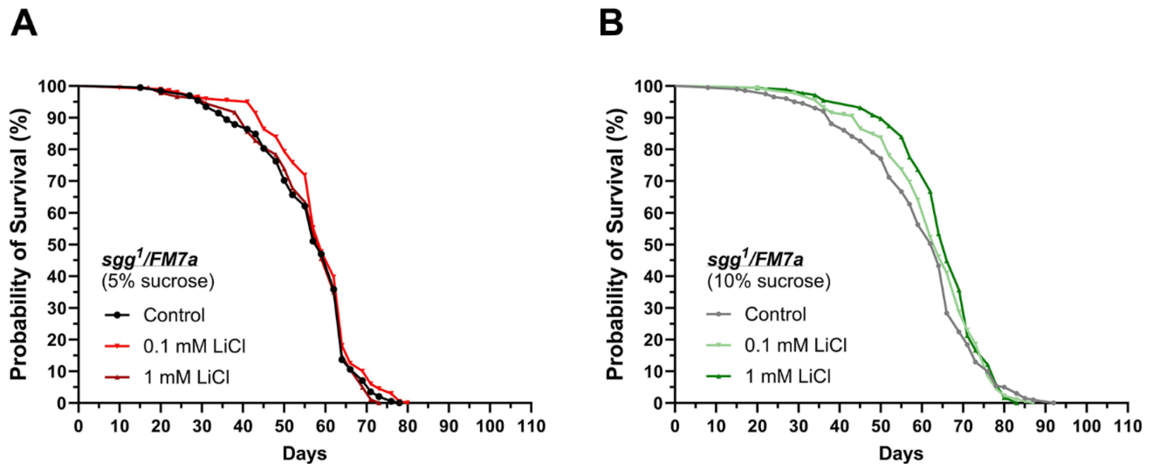

Supplementary Figure 1. *sgg*^{1}*FM7a* survival is not affected by dietary lithium added to a 5% or 10% diet. Survival curves of female *sgg*^{1}*FM7a* flies receiving 0.1 or 1 mM LiCl were not affected when supplemented to a 5% sucrose diet (**A**) or a 10% sucrose diet (**B**). Survival curves were compared using the Log-Rank Mantel-Cox test ($p \leq 0.05$).

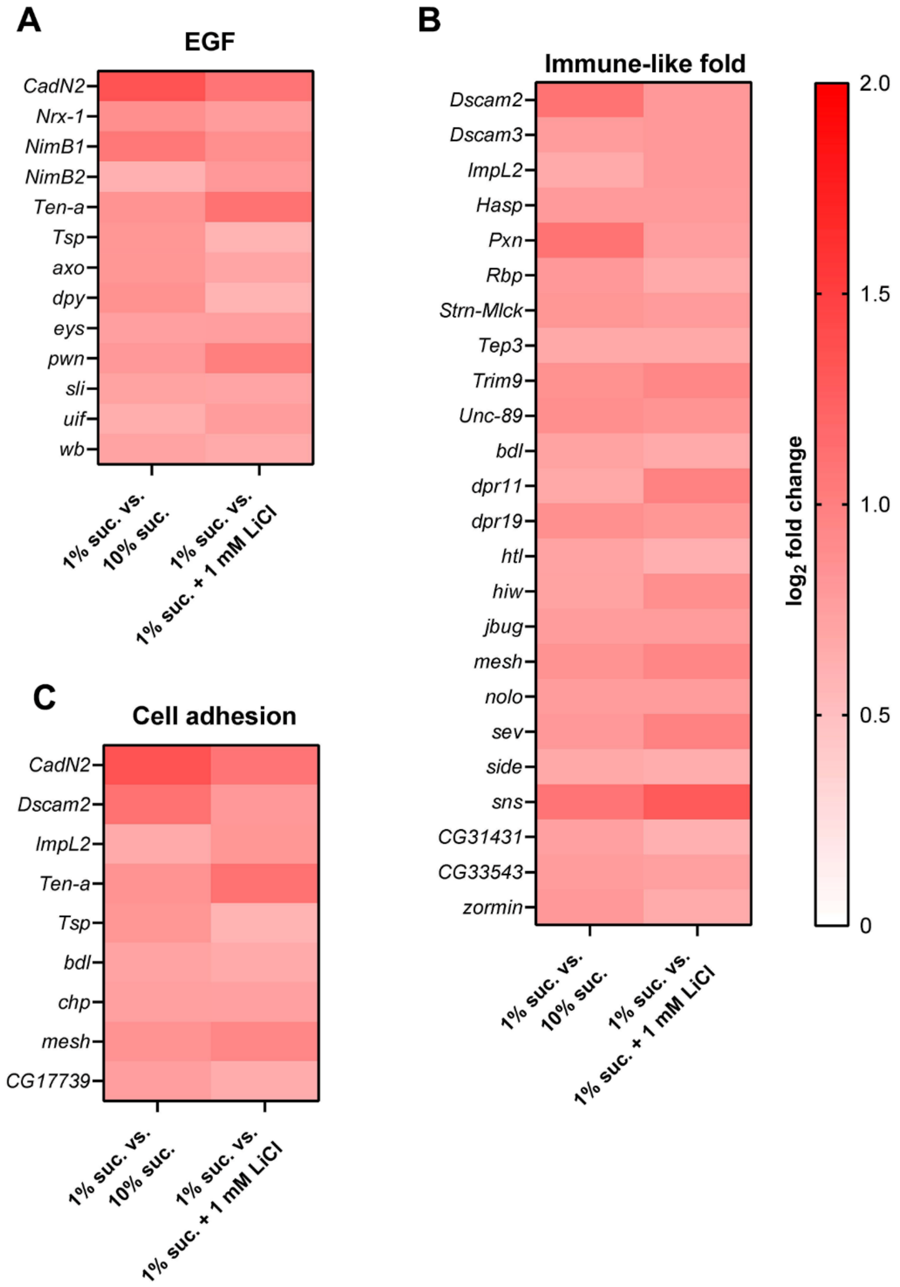

Supplementary Figure 2. Lithium and sucrose alter transcript levels of genes involved with EGF, Immune-like fold and cell adhesion in female *w*[1118]. Functional annotation of co-regulated genes differentially expressed in response to either elevating the dietary sucrose content by a factor of 10 or by supplementing 1 mM LiCl to the 1% sucrose diet (as listed in Supplementary Table 3.) revealed significant clusters regarding the following terms: epidermal growth factor (EGF) (**A**), immune-like fold (**B**) and cell adhesion (**C**) (Scale shows $\log_2$ fold changes of differentially expressed genes according to FDR $p \leq 0.05$).

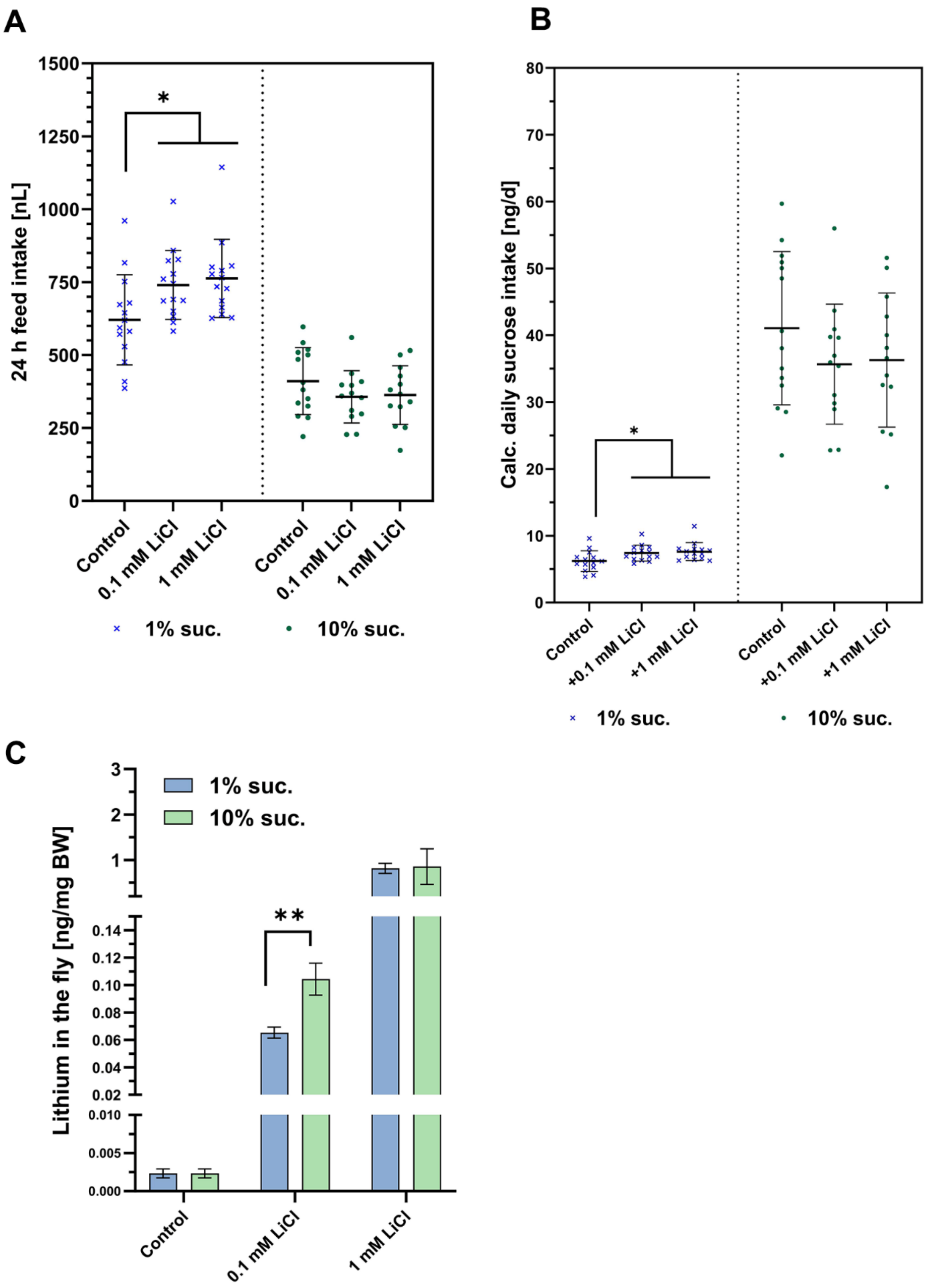

Supplementary Figure 3. Higher feed intake due to low dietary sugar did not translate into higher lithium status in female *w*[1118]. (**A**) The Excreta quantification assay revealed that lithium and sucrose both affect the feeding rate (Kruskal-Wallis test, *p ≤ 0.05). (**B**) Despite the higher feeding rate, the daily sugar intake was still significantly lower in flies receiving 1% sucrose compared to flies administered 10% sucrose diets (Kruskal-Wallis test, ****p ≤ 0.0001). (**C**) Higher feeding rates did not translate into a higher lithium status in flies receiving 1% sucrose (Unpaired t-test, **p ≤ 0.01). Data is given as mean ± SD, n = 13 - 14 with each n comprising 20 female *w*[1118]).
