# Supplementary material for: Dietary sucrose determines the regulatory activity of lithium on gene expression and lifespan in Drosophila melanogaster
Source: Aging (Albany NY). 2024 Jun 10;16(11):9309–33. doi: 10.18632/aging.205933 (PMC11210232; doi:10.18632/aging.205933)
Supplement: Supplementary Tables [file aging-16-205933-s002.pdf]

SUPPLEMENTARY TABLES

Supplementary Table 1. Key resource table.

Reagent or resource	Source	Identifier
**Modified Organisms**		
w^{1118}	Bloomington Drosophila Stock Center	Cat#5905
sgg1/FM7a/Dp(1;2;Y)w+	Bloomington Drosophila Stock Center	Cat#4095
**Antibodies**		
Anti-GSK-3 clone 4G-1E	Merck	Cat#05-412
Phospho-GSK-3β (Ser9) Rabbit mAb	Cell signaling Technology	Cat#5558
Immun-Star goat anti-mouse (GAM)-HRP conjugate	Bio-Rad Laboratories	Cat#1705047
Immun-Star goat anti-rabbit	Bio-Rad Laboratories	Cat#1705046
**Chemicals and kits**		
Inactive dry yeast	Genesee	Cat#62-107
Drosophila agar type 2	Genesee	Cat#66-103
D(+)-sucrose	Carl Roth	Cat#4621.2; CAS-ID: 57-50-1
Propionic Acid	Carl Roth	Cat#6026.1; CAS-ID: 79-09-4
Nipagin/Tegosept	Genesee	Cat# 20-258
EtOH absolute	VWR	Cat#20821.296; CAS-ID: 64-17-5
Lithium chloride	Merck	Cat#105679; CAS-ID: 7447-41-8
ROTI load loading dye	Carl Roth	Cat#K929.1
Pierce BCA Protein Assay Kit	Thermo Fisher Scientific	Cat#23225
Polyacrylamide (4 - 15%) precast gels	Bio-Rad Laboratories	Cat#4561083EDU
Methanol ≥99%	VWR	Cat#L13255.AP; CAS-ID: 67-56-1
Bovine serum albumin (BSA)	Carl Roth	Cat#T844.3; CAS-ID: 9048-46-8
Tween20	Merck	Cat#P9416-50ML; CAS-ID: 9005-64-5
SuperSignal Western Blot Substrate	Thermo Fisher Scientific	Cat#A43841
Triton X100	Merck	Cat#X100-5ML; CAS-ID: 9036-19-5
Infinity triglycerides liquid stable reagent	Thermo Fisher Scientific	Cat#TR22421
Glucose oxidase/peroxidase-linked (GOD-PAP) kit	Dialab	Cat#D08220
Trehalase (taken from the Kit)	Megazyme	Cat#K-TREH
Amyloglucosidase (taken from the Kit)	Megazyme	Cat#E-AMGDFNG-20ML
Trehalose dihydrate	Carl Roth	Cat#5151.3; CAS-ID: 6138-23-4
Glycogen	Sigma Aldrich	Cat#10901393001
peqGOLD TriFast	VWR	Cat#30-2010
Diethylpyrocarbonat (DEPC)	Merck	Cat#D5758; CAS-ID: 1609-47-8
DNA-free DNA Removal Kit	Thermo Fisher Scientific	Cat#AM1906
TruSeq Stranded mRNA Preb kit	Illumina	Cat#20020595
sodium hypochlorite (5-10 %)	Carl Roth	Cat#6846.1; CAS-ID: 7681-52-9
DNeasy Blood & Tissue Kit	Qiagen	Cat#69504
qPCRBIO SyGreen kit	PCR Biosystems	Cat#PB25.11-03
**Online tools and software**		
Image Lab 5.0	Bio-Rad Laboratories	https://www.bio-rad.com
CLC Genomics Workbench version 9.5.2	Qiagen	https://digitalinsights.qiagen.com/
Database for Annotation, Visualization and Integrated Discovery (DAVID)	National Institutes of Health, USA	https://david.ncifcrf.gov/tools.jsp
GraphPad Prism 10.0.2	GraphPad software	https://www.graphpad.com/
Pscan Web 1.6	Bio.tools, supported by ELIXIR	http://159.149.160.88/pscan/
CLC Genomics Workbench 21.0.3	Qiagen	https://digitalinsights.qiagen.com/
**Technical equipment**		
TissueLyser II	Qiagen	N/A
trans-blot turbo transfer system	Bio-Rad Laboratories	Cat#17001918

NovaSeq 6000 Sequencing System	Illumina	https://emea.illumina.com
Invitrogen Qubit 4 fluorometer	Thermo Fisher Scientific	Cat#15723679
GeneAmp PCR system 9700	Applied Biosystems	Cat#4426825

Supplementary Table 2. Transcription factor binding site analysis.

Matrix ID	Symbol	Name	P-value
MA0535.1	**Mad**	Mothers against Dpp	3.54 E-16
MA1700.1	**Clamp**	Chromatin-linked adaptor for MSL proteins	6.41 E-16
MA0205.2	**Trl**	Trithorax-like	8.38 E-13
MA0213.1	**brk**	brinker	5.92 E-09
MA0449.1	**h**	hairy	1.37 E-07
MA0185.1	**Deaf1**	Deformed epidermal autoregulatory factor-1	1.46 E-07
MA0443.1	**btd**	buttonhead	3.11 E-06
MA0247.2	**tin**	tinman	4.54 E-06
MA0456.1	**opa**	odd paired	1.16 E-05
MA0255.1	**z**	zeste	1.22 E-05
MA0016.1	**usp**	ultraspiracle	4.02 E-05
MA0450.1	**hkb**	huckebein	0.0003
MA0023.1	**dl(var.2)**	dorsal	0.00052
MA0917.1	**gcm2**	glial cells missing 2	0.0012
MA0193.1	**schlank**	schlank	0.0042
MA0086.2	**sna**	snail	0.02
MA1461.1	**sv**	shaven	0.03

Co-regulated genes of the overlap in Figure 5 were analyzed for the prevalence of transcription factor binding sites, revealing high frequency of sequences assigned to Mothers against Dpp (Mad) in female w^{1118}.

Supplementary Table 3. Functional annotation chart.

Category	Term	Count	%	p-value	Benjamini
GOTERM_CC_DIRECT	chorion	20	61.7	1.4 E-18	2.2 E-16
GOTERM_CC_DIRECT	integral component of plasma membrane	47	45.3	1.1 E-11	9.1 E-10
UP_SEQ_FEATURE	COMPBIAS:Polar residues	214	39.6	3.4 E-09	2.1 E-06
UP_SEQ_FEATURE	COMPBIAS:Pro residues	53	34.1	4.8 E-08	1.5 E-05
UP_KW_CELLULAR_COMPONENT	Membrane	161	31.8	2.2 E-06	4.8 E-05
UP_KW_CELLULAR_COMPONENT	Secreted	31	30.7	1.8 E-05	2.0 E-04
GOTERM_CC_DIRECT	extracellular region	41	26.1	4.1 E-06	2.2 E-04
INTERPRO	Immunoglobulin-like fold	23	11.2	4.9 E-07	2.7 E-04
UP_KW_BIOLOGICAL_PROCESS	Vision	11	11.2	5.6 E-06	2.8 E-04
UP_KW_DOMAIN	Signal	187	10.4	2.3 E-05	4.9 E-04
GOTERM_CC_DIRECT	synapse	19	10	1.2 E-05	4.9 E-04
UP_KW_PTM	Disulfide bond	53	8.7	4.5 E-05	5.0 E-04
INTERPRO	Immunoglobulin subtype 2	16	8.7	2.5 E-06	5.9 E-04
INTERPRO	Immunoglobulin subtype	16	8.3	3.5 E-06	5.9 E-04
INTERPRO	Epidermal growth factor-like domain	13	7.4	4.5 E-06	5.9 E-04
INTERPRO	Immunoglobulin I-set	13	6.8	5.2 E-06	5.9 E-04
UP_KW_MOLECULAR_FUNCTION	Retinal protein	5	6.6	1.1 E-05	6.2 E-04
UP_KW_MOLECULAR_FUNCTION	Photoreceptor protein	5	4.9	2.5 E-05	7.1 E-04
INTERPRO	Immunoglobulin-like domain	17	4.2	7.7 E-06	7.2 E-04
UP_SEQ_FEATURE	COMPBIAS:Basic and acidic residues	150	4	3.7 E-06	7.8 E-04

INTERPRO	Concanavalin A-like lectin/glucanase, subgroup	11	3.6	1.6 E-05	1.3 E-03
UP_SEQ_FEATURE	TRANSMEM:Helical	145	3.4	8.5 E-06	1.3 E-03
UP_KW_PTM	Glycoprotein	49	3.4	2.6 E-04	1.4 E-03
SMART	EGF	13	3.4	1.0 E-05	1.6 E-03
SMART	IGc2	16	3.4	2.7 E-05	2.0 E-03
SMART	IG	16	3.4	3.8 E-05	2.0 E-03
UP_SEQ_FEATURE	CARBOHYD:N-linked (GlcNAc...) asparagine	39	3.2	1.9 E-05	2.2 E-03
UP_SEQ_FEATURE	TOPO_DOM:Extracellular	32	2.8	2.1 E-05	2.2 E-03
INTERPRO	Fibronectin, type III	11	2.8	3.4 E-05	2.3 E-03
INTERPRO	Visual pigments (opsins) retinal binding site	5	2.8	3.7 E-05	2.3 E-03
GOTERM_CC_DIRECT	integral component of membrane	123	2.3	1.1 E-04	3.6 E-03
UP_SEQ_FEATURE	DOMAIN:EGF-like	11	2.3	6.0 E-05	5.4 E-03
GOTERM_BP_DIRECT	multicellular organism development	10	2.3	9.7 E-06	8.5 E-03
GOTERM_CC_DIRECT	neuromuscular junction	11	2.3	3.7 E-04	9.8 E-03
UP_KW_LIGAND	Chromophore	5	2.3	4.8 E-04	1.1 E-02
UP_SEQ_FEATURE	COMPBIAS:Basic residues	41	2.1	1.7 E-04	1.3 E-02
UP_SEQ_FEATURE	DOMAIN:Ig-like	15	2.1	1.8 E-04	1.3 E-02
UP_KW_BIOLOGICAL_PROCESS	Cell adhesion	9	1.9	6.4 E-04	1.6 E-02
INTERPRO	Laminin G domain	6	1.9	2.9 E-04	1.6 E-02
SMART	LamG	6	1.9	4.3 E-04	1.7 E-02
UP_SEQ_FEATURE	TOPO_DOM:Cytoplasmic	35	1.9	2.7 E-04	1.7 E-02
UP_SEQ_FEATURE	DOMAIN:Fibronectin type-III	9	1.9	3.0 E-04	1.7 E-02
UP_KW_DOMAIN	Immunoglobulin domain	7	1.9	1.7 E-03	1.8 E-02
KEGG_PATHWAY	Lysosome	9	1.5	3.8 E-04	1.8 E-02
SMART	FN3	9	1.5	5.9 E-04	1.8 E-02
UP_SEQ_FEATURE	REGION:Disordered	291	1.3	4.0 E-04	2.1 E-02
GOTERM_MF_DIRECT	G-protein coupled photoreceptor activity	5	1.3	6.3 E-05	2.3 E-02
GOTERM_BP_DIRECT	visual perception	10	1.3	6.3 E-05	2.7 E-02
GOTERM_CC_DIRECT	cytoskeleton of presynaptic active zone	4	1.1	1.6 E-03	3.3 E-02
GOTERM_CC_DIRECT	presynaptic active zone	6	1.1	1.7 E-03	3.3 E-02
GOTERM_BP_DIRECT	protein-chromophore linkage	5	1.1	1.4 E-04	3.7 E-02
GOTERM_BP_DIRECT	axon guidance	16	1.1	1.8 E-04	3.7 E-02
GOTERM_BP_DIRECT	chorion-containing eggshell formation	7	1.1	2.1 E-04	3.7 E-02
GOTERM_BP_DIRECT	neuromuscular synaptic transmission	9	1.1	2.6 E-04	3.8 E-02
UP_KW_CELLULAR_COMPONENT	Synapse	9	0.8	6.2 E-03	4.5 E-02
SMART	LamG	6	1.9	4.3 E-04	1.7 E-02
UP_SEQ_FEATURE	TOPO_DOM:Cytoplasmic	35	1.9	2.7 E-04	1.7 E-02
UP_SEQ_FEATURE	DOMAIN:Fibronectin type-III	9	1.9	3.0 E-04	1.7 E-02
UP_KW_DOMAIN	Immunoglobulin domain	7	1.9	1.7 E-03	1.8 E-02
KEGG_PATHWAY	Lysosome	9	1.5	3.8 E-04	1.8 E-02
SMART	FN3	9	1.5	5.9 E-04	1.8 E-02
UP_SEQ_FEATURE	REGION:Disordered	291	1.3	4.0 E-04	2.1 E-02
GOTERM_MF_DIRECT	G-protein coupled photoreceptor activity	5	1.3	6.3 E-05	2.3 E-02
GOTERM_BP_DIRECT	visual perception	10	1.3	6.3 E-05	2.7 E-02
GOTERM_CC_DIRECT	cytoskeleton of presynaptic active zone	4	1.1	1.6 E-03	3.3 E-02
GOTERM_CC_DIRECT	presynaptic active zone	6	1.1	1.7 E-03	3.3 E-02
GOTERM_BP_DIRECT	protein-chromophore linkage	5	1.1	1.4 E-04	3.7 E-02
GOTERM_BP_DIRECT	axon guidance	16	1.1	1.8 E-04	3.7 E-02
GOTERM_BP_DIRECT	chorion-containing eggshell formation	7	1.1	2.1 E-04	3.7 E-02
GOTERM_BP_DIRECT	neuromuscular synaptic transmission	9	1.1	2.6 E-04	3.8 E-02
UP_KW_CELLULAR_COMPONENT	Synapse	9	0.8	6.2 E-03	4.5 E-02

Genes of the overlap in Figure 5 were analyzed for the functional annotation (female w1118). Only statistically significant clusters are given (Benjamini $p < 0.05$).
